# miR-203 inhibits the traumatic heterotopic ossification by targeting Runx2

**DOI:** 10.1038/cddis.2016.325

**Published:** 2016-10-27

**Authors:** Bing Tu, Shen Liu, Bo Yu, Jing Zhu, Hongjiang Ruan, Tingting Tang, Cunyi Fan

**Affiliations:** 1 Department of Orthopaedic Surgery, Shanghai Jiaotong University Affiliated Sixth People's Hospital, Shanghai, China; 2 Department of Orthopaedics, Affiliated Hospital of Shandong University of Traditional Chinese Medicine, Jinan, China; 3 Department of Anatomy, School of Basic Medicine, Shanghai University of Traditional Chinese Medicine, Shanghai, China; 4 Shanghai Key Laboratory of Orthopedic Implants, Department of Orthopedic Surgery, Shanghai Ninth People's Hospital, Shanghai Jiao Tong University School of Medicine, Shanghai, China

## Abstract

Emerging evidence has indicated that dysregulated microRNAs (miRNAs) have an important role in bone formation. However, the pathophysiological role of miRNAs in traumatic heterotopic ossification (HO) remains to be elucidated. Using gene expression profile analyses and subsequent confirmation with real-time PCR assays, we identified the decreased expression of miRNA-203 (miR-203) and increased expression of Runx2 as responses to the development of traumatic HO. We found that miR-203 expression was markedly higher in primary and recurrent HO tissues than in normal bones. The upregulation of miR-203 significantly decreased the level of Runx2 expression, whereas miR-203 downregulation increased Runx2 expression. Mutation of the putative miR-203-binding sites in Runx2 mRNA abolished miR-203-mediated repression of Runx2 3'-untranslated region luciferase reporter activity, indicating that Runx2 is an important target of miR-203 in osteoblasts. We also found that miR-203 is negatively correlated with osteoblast differentiation. Furthermore, *in vitro* osteoblast activity and matrix mineralization were promoted by antagomir-203 and decreased by agomir-203. We showed that miR-203 suppresses osteoblast activity by inhibiting the *β*-catenin and extracellular signal-regulated kinase pathways. Moreover, using a tenotomy mouse HO model, we found an inhibitory role of miR-203 in regulating HO *in vivo*; pretreatment with antagomiR-203 increased the development of HO. These data suggest that miR-203 has a crucial role in suppressing HO by directly targeting Runx2 and that the therapeutic overexpression of miR-203 may be a potential strategy for treating traumatic HO.

Heterotopic ossification (HO) is a clinically devastating complication defined as the presence of lamellar bone in soft tissues, where bone does not normally occur.^1^ Although HO can arise from fibrodysplasia ossificans progressiva (FOP), HO is typically found following fractures and dislocations, burns, traumatic brain injury and operative procedures.^2^ HO often appears within the soft tissue surrounding the joints, leading to joint stiffness, nerve entrapment and persistent pain. Similar to normal skeletal morphogenesis, HO can arise through either an intramembranous or endochondral process, suggesting that multiple mechanisms are involved.^3^ However, HO occurs predominantly through an intramembranous process, and ectopic osteoblasts differentiate from mesenchymal progenitors independently of chondrocytes in this type of disorder.^4^ Although the pathogenesis of HO remains unclear, several factors that may contribute to this process have been identified. It is believed that the inappropriate proliferation and differentiation of osteoblasts contributes to the bone formation.

MicroRNAs (miRNAs) are a family of short, single-stranded noncoding RNAs that repress the expression of target genes through either mRNA degradation or translational inhibition.^5, 6^ A wide spectrum of miRNAs has been found to regulate the expression of osteogenic differentiation marker genes and osteogenesis.^7, 8^ However, most of these miRNAs have been investigated only *in vitro* or in normal skeletogenesis. Their functional roles in the pathophysiological mechanisms responsible for HO remain to be established. Runx2, a runt-related transcription factor, has a pivotal role in osteoblast differentiation and bone formation.^9^ Previous studies have demonstrated that Runx2 is aberrantly expressed in the ossification of soft tissue, including ligaments and tendons.^10, 11^ The inhibition of Runx2 by RNA interference suppresses osteogenesis and prevents ectopic bone formation, thus providing a basis for Runx2 as a potential therapeutic target in preventing HO.^12, 13^

In this study, we screened for the expression of miRNAs in traumatic HO specimens from patients with fractures and observed that miR-203 was negatively correlated with the formation of HO. Our study demonstrated that miR-203 participates in the inhibition of osteoblast differentiation and traumatic HO in a mouse model. We identified Runx2, the master regulator of osteoblast differentiation, as a direct target of miR-203. Our findings further demonstrated that the therapeutic overexpression of miR-203 in osteoblasts may lead to decreased Runx2 expression, which coincides with the inhibition of HO in tenotomy mice. Thus, our study provides a new mechanism and a novel therapeutic target for the development of traumatic HO.

## Results

### miR-203 and Runx2 expression are inversely correlated in HO patients

**Figure 1:**
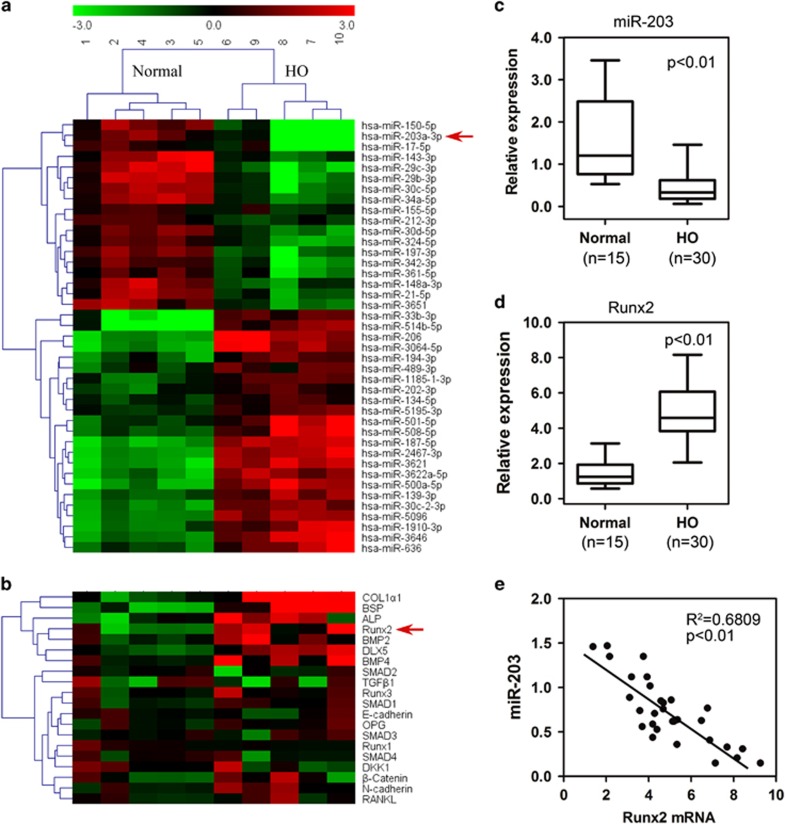
Expression of miR-203 is negatively correlated with Runx2 in HO patients. (**a**) Microarray profiling results of dysregulated miRNAs in HO and normal bone samples from patients. (**b**) Heat map depicting the relatedness of the gene expression profiles of normal bones and HO tissues. (**c**) The miR-203 level is decreased in the HO tissues (*n*=30) compared with the normal bone samples (*n*=15). (**d**) The Runx2 mRNA level is increased in the HO tissues. (**e**) A significant inverse correlation is observed between the miR-203 and Runx2 expression levels in the HO tissues (*n*=30)

To determine the mechanism of developing HO, normal bone and HO tissues from patients were collected to identify dysregulated miRNAs by performing an miRNA microarray analysis (Figure 1a). Among the miRNAs, miR-203 exhibited a substantial difference in expression between the two groups, with expression levels approximately 10 times higher in HO specimens than in normal bones. To investigate the gene expression profile in HO, we generated a heat map that showed genes expressed in both normal bone and HO samples. We noted an increased expression of Runx2 and osteogenic marker genes in the HO samples (Figure 1b). Real-time PCR showed that the expression of several miRNAs including miR-203, miR-29c, miR-30c, miR-34a and miR-148a was substantially reduced in the HO samples (Supplementary Figure 1A). However, only expression of miR-203, not the other miRNAs, was negatively correlated with the expression of Runx2 in the HO samples (Supplementary Figure 1B). The HO tissues had significantly decreased levels of mature miR-203 compared with normal bone tissues (Figure 1c). In addition, using real-time PCR analysis, we analyzed Runx2 mRNA expression in 15 normal bone tissue samples and 30 HO tissues. We found that Runx2 mRNA expression was significantly upregulated in HO tissue samples (Figure 1d). Using non-parametric tests, we found a significant inverse correlation between Runx2 mRNA and miR-203 expression in the HO samples (*R*^2^=0.6809; Figure 1e).

### Runx2 is a direct target of miR-203

**Figure 2:**
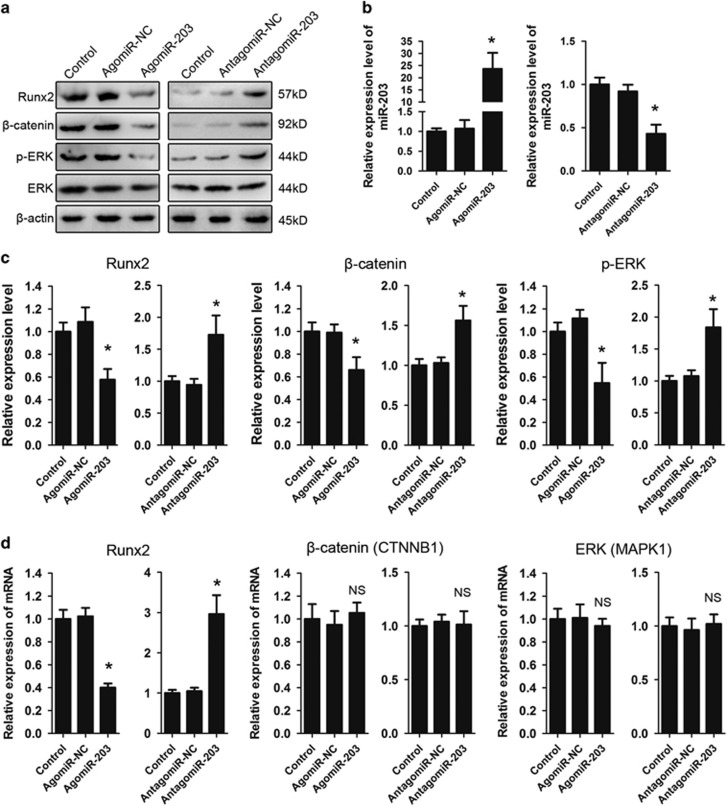
miR-203 targets Runx2 and inhibits the *β-*catenin and ERK pathways. (**a**) hFOB1.19 cells were transfected with agomiR-203 or antagomiR-203. Western blot analysis of the relative levels of Runx2, *β*-catenin, p-ERK and ERK protein expression at 48 h. (**b**) Real-time PCR analysis of the relative levels of miR-203 expression in hFOB1.19 cells transfected with 10 *μ*M agomiR-203, antagomiR-203 or their NCs. (**c**) Densitometric analysis of Runx2, *β*-catenin and p-ERK in the hFOB1.19 cells transfected with agomiR-203 or antagomiR-203. (**d**) Real-time PCR analysis of Runx2, *β*-catenin (CTNNB1 gene) and ERK (MAPK1 gene) in the hFOB1.19 cells transfected with agomiR-203 or antagomiR-203. The data are representative of three independent experiments. *P<0.01; NS, not significant

Previous studies have suggested that *β*-catenin and extracellular signal-regulated kinase (ERK) utilize important pathways associated with Runx2 and participate in bone formation.^14, 15^ The effects of miR-203 transfection on Runx2, *β*-catenin and p-ERK levels were analyzed by western blotting (Figure 2a). No significant proliferation difference was found after the transfection with agomiR-203 or antagomiR-203 (Supplementary Figure 2). The increased expression of miR-203 upon transfection was confirmed with a real-time PCR assay (Figure 2b), and the ectopic expression of miR-203 significantly reduced the levels of Runx2, *β*-catenin and p-ERK 48 h after transfection as shown by densitometric analysis (Figures 2a and c, left panels). Conversely, knockdown with antagomiR-203 increased the levels of Runx2, *β*-catenin and p-ERK protein (Figures 2a and c, right panels). Transfection with agomiR-203 or antagomiR-203 had a similar effect on Runx2 mRNA expression. However, miR-203 did not affect *β*-catenin (CTNNB1 gene) and ERK (MAPK1 gene) mRNA expression (Figure 2d). To investigate whether miR-203 regulated *β*-catenin and ERK signaling through Runx2, hFOB1.19 cells were co-transfected with a Runx2 siRNA and antagomiR-203. We found that Runx2 knockdown inhibited the activation of ERK and *β*-catenin by antagomiR-203 (Supplementary Figure 3A). Then hFOB1.19 cells were co-transfected with a Runx2 overexpression plasmid and agomiR-203. Our results showed that re-expression of Runx2 rescued the inhibitory effects of agomiR-203 (Supplementary Figure 3B). These data together suggested that miR-203 inhibited the ERK and *β*-catenin signaling in a Runx2-dependent manner.

**Figure 3:**
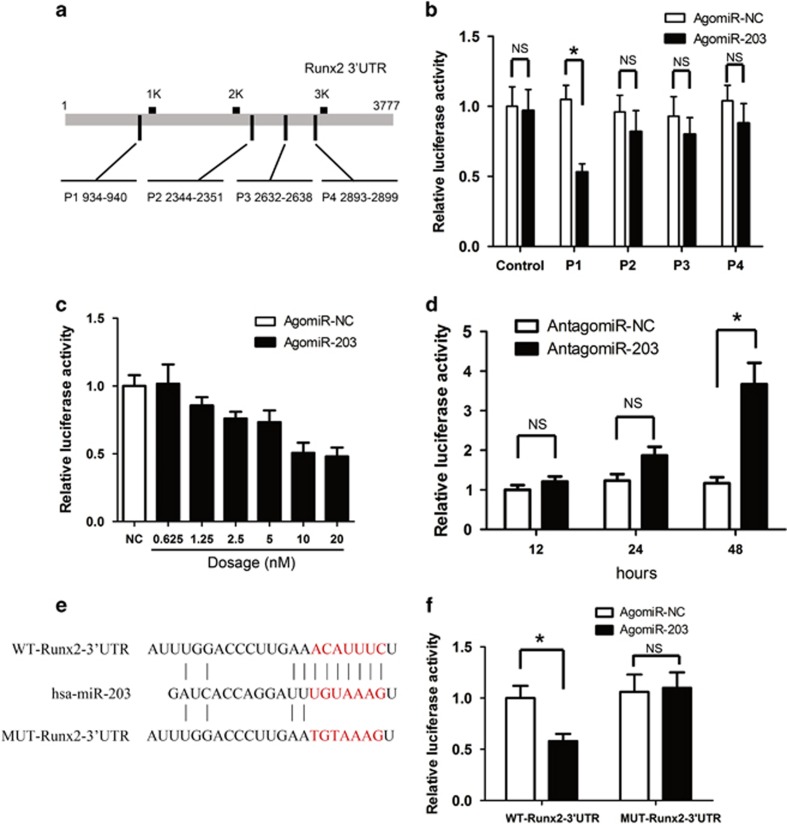
miR-203 targets the MREs in the 3'-UTR of Runx2. (**a**) Sequence alignment of miR-203 with the putative MREs in the 3'-UTR of the human Runx2 gene. (**b**) Regulation of the Runx2 reporter gene expression by agomiR-203. (**c**) Dose-dependent suppression of pGL3-P1 by miR-203. (**d**) Effects of antagomiR-203 on the luciferase activity of pGL3-P1. (**e**) A schematic diagram illustrating the design of luciferase reporters with the WT-Runx2 3'-UTR and mutant Runx2 3'-UTR. (**f**) hFOB1.19 cells were transfected with a luciferase reporter carrying the WT or MUT 3'-UTR of the Runx2 gene (WT-Runx2-3'-UTR or MUT-Runx2-3'-UTR) and co-transfected with agomiR-203 or agomiR-NC. The effects of miR-203 on the reporter genes were determined at 48 h after transfection. The data are representative of three independent experiments. The data are shown as the means±S.D. *P<0.01; NS, not significant

Using Targetscan and miRBase searches, we identified four miRNA regulatory elements (MREs) (positions 1–4; P1–P4) for miR-203 in the 3'-untranslated region (UTR) of Runx2 mRNA (Figure 3a). We individually cloned these four partial 3'-UTRs into the pGL3 reporter vector downstream of the luciferase open-reading frame. We found that cells transfected with the P1 luciferase reporter plus miR-203 exhibited significantly less luciferase activity (Figure 3b). Using this luciferase reporter system, we found that the inhibition of luciferase activity by agomiR-203 was dose dependent (Figure 3c). To examine the specificity of miR-203, we showed that the miRNA inhibitor antagomiR-203 specifically abolished the luciferase activity (Figure 3d). A Runx2 3'-UTR luciferase reporter that contains mutated sequences of the miR-203-binding sites (MUT-Runx2-3'-UTR) was constructed, and hFOB1.19 cells were transfected with agomiR-203 or agomiR-NC (Figure 3e). The overexpression of miR-203 suppressed the luciferase activity of the Runx2 3'-UTR reporter genes, whereas mutation within the sequences abolished this repression (Figure 3f).

### miR-203 inhibits osteoblastic differentiation by targeting Runx2

**Figure 4:**
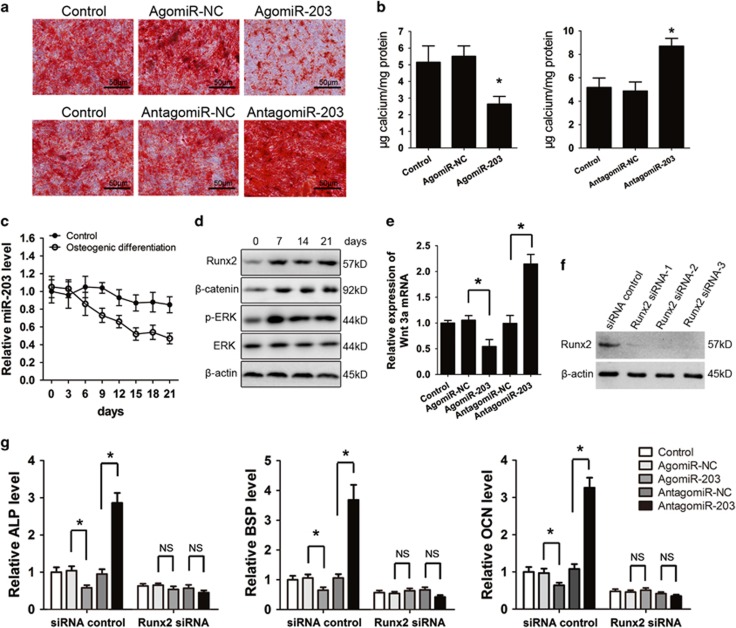
miR-203 inhibits the osteogenic differentiation of osteoblasts. The hFOB1.19 cells transfected with agomiR-203, antagomiR-203, or their controls were cultured in OM for 3 weeks. (**a**) Representative images of alizarin red S staining. (**b**) Quantitative analysis of matrix mineralization. (**c**) Changes in the miR-203 level during osteogenesis in hFOB1.19 cells were detected by RT-PCR analysis. (**d**) The expression of the Runx2, *β*-catenin, p-ERK and ERK proteins during osteogenesis was detected by western blot analysis. (**e**) The hFOB1.19 cells were transfected with agomiR-203, antagomiR-203 or their controls for 48 h, relative mRNA level of Wnt 3a was detected by real-time PCR. (**f**) The knockdown efficiency of Runx2 siRNA was confirmed by western blot analysis. (**g**) Real-time PCR analysis of OCN, ALP and BSP mRNA in hFOB1.19 cells after co-transfecting Runx2 siRNA with agomiR-203, antagomiR-203 or their NCs for 3 days. All of the data are expressed as the means±S.D. from three independent experiments. *P<0.01; NS, not significant

To investigate the role of miR-203 during osteoblastic differentiation, hFOB1.19 cells were transfected with agomiR-203 or antagomiR-203 to overexpress or silence the miRNA, respectively, and then the cells were cultured in osteogenesis induction medium (OM). Alizarin red staining revealed that the overexpression of miR-203 inhibited the osteogenic differentiation of the osteoblast cells, whereas the silencing of miR-203 promoted the osteogenic differentiation process (Figures 4a and b). Alkaline phosphatase (ALP) and osteocalcin (OCN) mRNA levels were upregulated by antagomir-203 and downregulated by agomir-203 compared with treatment with the negative controls (NCs) for these constructs (Supplementary Figures 4A and 4B). The ALP activity and supernatant protein concentrations of bone sialoprotein (BSP) were substantially lower in the agomir-203 treatment group and were markedly higher in the antagomir-203 treatment group (Supplementary Figures 4C and 4D). To clarify the expression profile of miR-203 during osteoblast differentiation, hFOB1.19 cells were cultured in OM for 21 days. We found that the expression of miR-203 decreased gradually during osteogenesis (Figure 4c). In addition, the Runx2, *β*-catenin and p-ERK protein levels were strongly increased from the onset of osteoblast differentiation and were maintained at a high level until day 21 (mineralization stage) (Figure 4d). Transfection with agomiR-203 decreased the expression level of Wnt 3a, whereas antagomiR-203 treatment enhanced expression of Wnt 3a (Figure 4e). To determine whether miR-203-regulated osteoblast differentiation is Runx2 dependent, we used a Runx2 siRNA to verify the effect of Runx2 on osteoblast differentiation (Figure 4f). We then determined the intracellular miR-203 levels by real-time PCR analysis after treatment with agomir-203 or antagomir-203 (Supplementary Figure 4E). We found that transfection with Runx2 siRNA significantly reduced the mRNA levels of the osteogenic marker genes ALP, BSP and OCN. Furthermore, the effects of miR-203 were completely blocked by Runx2 siRNA transfection, suggesting that the function of miR-203 in osteoblast differentiation is Runx2 dependent (Figure 4g).

### Clinical HO samples exhibit elevated Runx2 expression and activated *β*-catenin and ERK signaling

**Figure 5:**
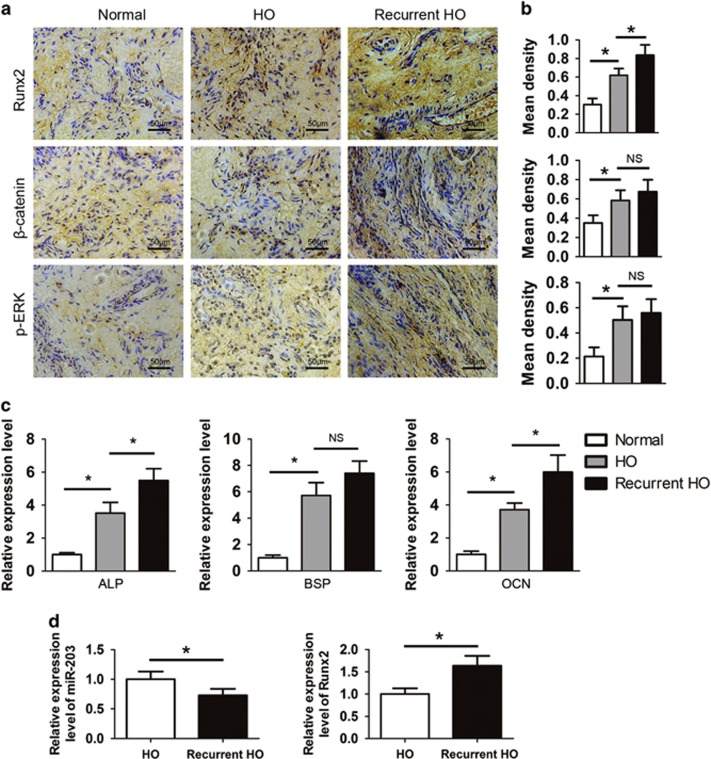
Clinical HO specimens exhibit the overexpression of Runx2 and activated *β*-catenin and ERK signaling. (**a**) Normal bones (*n*=15) and primary (*n*=30) and recurrent HO tissues (*n*=7) were decalcified. The expression of Runx2, *β*-catenin and p-ERK was assayed by immunohistochemistry. (**b**) Quantification of Runx2, *β*-catenin and p-ERK expression. (**c**) Total RNA was isolated from normal bones and primary and recurrent HO tissues. The expression of ALP, BSP and OCN was examined with real-time PCR assays. (**d**) Total RNA was isolated from primary HO and recurrent HO tissues. The relative miR-203 and Runx2 levels were detected by real-time PCR assays. *P<0.01; NS, not significant

HO lesions were resected from elbow trauma patients, and normal bones were used as a control. All bones were demineralized and subjected to an immunohistochemistry assay. Increased expression of Runx2, *β*-catenin and p-ERK was observed in the HO tissues compared with normal bones. We observed a greater Runx2 expression level in recurrent HO than in primary HO. However, there was no significant difference in *β*-catenin and p-ERK expression levels between the primary and recurrent HO groups (Figures 5a and b). Next, we measured the gene expression of osteogenic markers in the HO samples. The real-time PCR results indicated that the ALP, BSP and OCN mRNA levels were increased in the HO tissues. The expression levels of ALP and OCN were markedly higher in the recurrent HO group, whereas BSP expression exhibited no significant difference (Figure 5c). Furthermore, we compared the recurrent HO tissues with primary HO tissues. We observed that the miR-203 expression level was lower and the Runx2 expression level was higher in the recurrent samples (Figure 5d).

### miR-203 inhibits the development of HO *in vivo*

**Figure 6:**
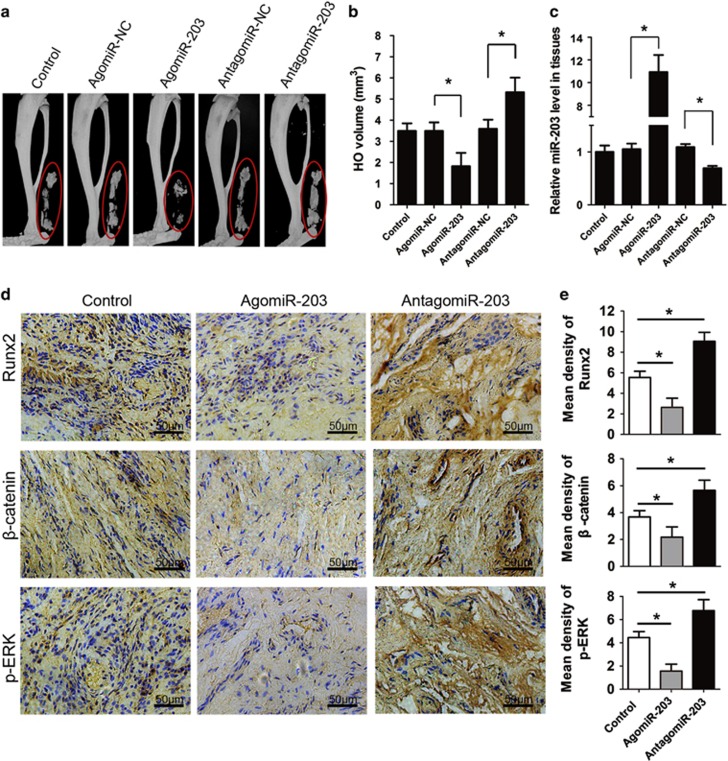
Therapeutic overexpression of miR-203 inhibits HO *in vivo*. (**a**) The Achilles tendon of the mice was divided at its midpoint with a surgical knife to generate a traumatic HO model. Then the mice were injected weekly at the lesion with agomiR-203, antagomiR-203, or their NCs (PBS was used as the control). Representative micro-CT reconstructed images of HO in the control, agomiR-NC, agomiR-203, antagomiR-NC and antagomiR-203 mice. (**b**) Quantification of the HO volumes. (**c**) Total RNA was isolated from the mice HO. The relative miR-203 levels were detected by real-time PCR assays. (**d**) HO tissues from the mice were decalcified, and the expression of Runx2, *β*-catenin and p-ERK was detected by immunohistochemistry at week 8. (**e**) Quantification of Runx2, *β*-catenin and p-ERK expression. *n*=10 per group. The data are shown as the means±S.D. *P<0.01

We further verified whether miR-203 had an essential role *in vivo*. Mice underwent a tenotomy to generate a traumatic HO model. To examine the therapeutic effects, the mice were subsequently treated with weekly injections of agomiR-203 or antagomiR-203 (80 mg/kg) at the lesion site; phosphate-buffered saline (PBS) served as a control. The mice treated with agomiR-203 exhibited significantly less HO than the NC group, whereas antagomiR-203 treatment increased the HO volumes (Figures 6a and b). Furthermore, the expression of miR-203 after the different *in vivo* treatments was determined by real-time PCR analysis. HO tissues from the mice treated with agomiR-203 had increased miR-203 levels, whereas treatment with antagomiR-203 resulted in decreased miR-203 levels (Figure 6c). The HO lesions were collected and subjected to an immunohistochemistry assay. We observed that the expression of Runx2, *β*-catenin and p-ERK was decreased after agomiR-203 treatment and was enhanced after antagomiR-203 treatment (Figures 6d and e).

## Discussion

This study found that miRNAs can be regulated under traumatic conditions and have essential roles in osteoblast differentiation and HO development. In our work, we demonstrated that miR-203 is involved in these processes by repressing Runx2 expression at the post-transcriptional level. Furthermore, our results suggest that the therapeutic overexpression of miR-203 in osteoblasts may reduce HO and even prevent this disorder.

It is believed that several miRNAs are important regulators of bone formation-related gene expression at the post-transcriptional level.^16, 17, 18^ However, it has not yet been determined whether miRNAs contribute to certain human skeletal disorders, such as HO. In our study, we found that decreased miR-203 levels accompanied the increased expression of osteoblast differentiation marker genes in patients with elbow HO. This observation provides clinical insight into the contribution of an miRNA to the pathophysiological regulation of the development of traumatic HO.

Previously, it has been reported that miR-203 expression is highly inhibited in many tumor cells,^19, 20, 21^ indicating that miR-203 has an important role in the initiation, progression and metastasis of tumors. However, few reports have focused on the role of miR-203 in the regulation of osteoblast function and the development of HO. Luo *et al.*^22^ has demonstrated that miR-203 represses primary myoblast proliferation and differentiation by targeting c-JUN and MEF2C. In our study, we found a mechanism by which miR-203 is involved in the regulation of the expression of the Runx2 protein in HO tissues. Runx2 activity facilitates bone formation by promoting osteoblast-specific gene expression.^23^ In addition, Runx2 accumulation can activate the *β-*catenin and ERK pathways, which promote osteogenic differentiation and bone formation.^24, 25^ In this study, Runx2, the principal transcriptional regulator of osteoblast differentiation, was activated during HO development and osteoblast differentiation.

Runx2 is a crucial bone-specific transcription factor in osteogenesis and bone formation. Runx2 regulates the expression of osteoblast-related genes by binding to their promoter regions.^26^ In addition, Runx2 has a 3.7-kb 3'-UTR that contains multiple regulatory elements. Therefore, Runx2 activation is likely modulated by several mechanisms in osteoblast differentiation. It has been reported that miR-320c/Runx2 axis regulates the balance between adipocytic and osteogenic differentiation of mesenchymal stem cells (MSCs).^27^ A recent research has shown that miR-103a suppresses the bone formation by targeting Runx2.^28^ Our data demonstrate that a change in the miR-203 level results in an altered expression of Runx2. Furthermore, our experimental evidence from *in vitro* and *in vivo* studies strongly suggests that Runx2 could be a functional target of miR-203 and may mediate its regulatory role in the development of HO.

The development of HO in soft tissues adjacent to the trauma sites was both a striking and unexpected finding. These conditions have been anecdotally associated with enhanced osteoblast activity.^29, 30^ The traumatic stimulation leads to the secretion of growth factors and cytokines, including TGF-*β*, BMPs, IGF, VEGF, PDGF and others, which are considered principal local regulators of osteogenesis.^31, 32^ The activation of intracellular signaling pathways by these growth factors may be attributed to specific miRNA dysregulation.^33^ *β*-Catenin and ERK1/2 are two of the most important osteoblast anabolic signaling pathways.^34, 35^ The inhibition of *β*-catenin or ERK1/2 prevents osteoblast differentiation from mesenchymal progenitors, suggesting that these two signaling pathways have critical roles in osteoblast differentiation.^14, 36^ In our study, we found that both the *β*-catenin and ERK pathways are significantly activated accompanied by osteogenic differentiation. These signaling pathways subsequently activate their downstream osteogenic marker genes, including Runx2. However, we found that the inhibition of these two signaling pathways does not affect the miR-203 expression level, indicating that the expression of miR-203 is not regulated by these two signaling pathways in osteoblast differentiation.

Several reports have suggested that Runx2 is directly involved in preventing HO.^13, 37^ In addition, it has been reported that Runx2 is one of the most important target genes regulated by the *β*-catenin and ERK pathways.^38^ Our data demonstrated that HO was significantly reduced when miR-203 levels were enhanced *in vivo*. After the administration of agomiR-203, the activation of the *β*-catenin and ERK signaling pathways was inhibited, and the Runx2 expression level was decreased. Therefore, we posit that the dysregulation of miR-203 leads to the overexpression of Runx2, which activates *β*-catenin and ERK signaling and promotes the development of HO. Future studies are required to better define the precise mechanism by which miR-203 and these transcription factors contribute to HO following trauma. The recurrence rate is relatively high after the primary HO is removed by surgery.^39^ Our data suggest that downregulated miR-203 may also have an important role in the recurrence of HO. We found that HO specimens from the recurrent patients exhibited a decreased expression of miR-203. As only seven recurrent HO patients were enrolled in our study, we did not find changes in the expression levels of BSP, *β*-catenin and p-ERK between the primary and recurrent patients. Given the limitations of this study it is difficult to conclude that high expression of *β*-catenin and p-ERK solely contribute to the recurrence of HO. However, the expression levels of these proteins in the recurrent group displayed increasing trends. These data indicate that activated *β*-catenin and p-ERK may have a role in the recurrent HO.

Our results provide a new paradigm for the role of miR-203 during the development of traumatic HO. We found that pharmacotherapy with agomiR-203 can potently diminish extraskeletal bone formation in the models of traumatic HO. Furthermore, our findings suggest that miR-203 functions by inhibiting its direct target Runx2 at the post-transcriptional level. Deciphering this mechanism is an important step toward unraveling the regulatory network that underlies traumatic HO, thereby bringing us closer to realizing the potential of using miRNA to prevent HO.

## Materials and Methods

### Patients and specimens

HO tissues were collected from 30 traumatic elbow patients who underwent surgical resection (6–8 months after injury) between January 2010 and December 2015, and normal bones were collected from 15 patients who underwent traumatic amputation (tibia, femur, radius and ulna). The median age of the patients was 32 years (range: 21–62 years). No significant difference was observed in the composition of these two groups regarding age or sex (*P*>0.05). Approval for this study was obtained from the ethics committee of Shanghai Jiao Tong University Affiliated Sixth People's Hospital, and written informed consent was obtained from the patients or their legal guardians. The experiment was performed in accordance with approved guidelines.

### RNA from tissues

Tissue samples were harvested from HO or amputation patients as described above. Soft tissues and epiphyses were removed; epiphyses were cut off, and diaphyses were flushed with a PBS solution to remove bone marrow and blood. All of the samples were shock frozen in liquid nitrogen, pulverized and dissolved in Trizol (Life Technologies, Carlsbad, CA, USA) for total RNA isolation.

### miRNA microarray assay

Small RNAs were isolated from the total RNA of HO and normal bone samples (five patients in each group). miRNAs were profiled using the Agilent Human miRNA Microarray V19.0 (Agilent, Santa Clara, CA, USA). The scanned images were analyzed with the Feature Extraction software 10.7.1.1 (Agilent) using default parameters to obtain background-subtracted and spatially detrended processed signal intensities as the raw data. The raw data were normalized with a quantile algorithm using Genespring 12.0 (Agilent). Probes for which at least 100% of the samples in any 1 of 2 conditions had flags in ‘Detected' were maintained.

### Gene chip microarray assay

Total RNA samples (five samples per group) were subjected to downstream microarray analysis. All of the hybridization experiments were performed using Affymetrix HG-U133 Plus 2.0 GeneChips (Affymetrix, Santa Clara, CA, USA) according to the manufacturer's recommendations. The raw data were normalized using Genespring GX11 Software (Agilent Technologies, Santa Clara, CA, USA) with default parameters (MAS5 Summarization Algorithm, median of all samples as the baseline transformation).

### Cell culture and transfection

Human preosteoblast hFOB1.19 cells were obtained from the Chinese Academy of Sciences (Shanghai, China). Cells were cultured in Dulbecco's modified Eagle's medium (DMEM; Hyclone, Logan, UT, USA) with 10% fetal bovine serum (FBS) and 0.3 mg/ml G418 (Sigma-Aldrich, St. Louis, MO, USA). After confluency was reached, the culture medium was changed to OM, which contained 10% FBS, 50 μM l-ascorbic acid, 10 mM glycerol-2-phosphate and 100 nM dexamethasone. AgomiR-203, antagomiR-203 and their NCs were purchased from RiboBio Co (Guangzhou, China). For the transfection of miRNAs, the cultured cells were transfected using Lipofectamine 2000 (Invitrogen, Carlsbad, CA, USA) according to the manufacturer's instructions. Runx2 siRNA and overexpression plasmid (RUNX2 CRISPR Activation Plasmid) were purchased from Santa Cruz Biotechnology (Santa Cruz, CA, USA).

### Cell proliferation assay

hFOB1.19 cells were seeded in 96-well plates at a density of 1 × 10^4^ per well and allowed to recover for 12 h. The proliferation of the cells was evaluated using a cell counting kit-8 (CCK-8, Dojindo, Japan) according to the as described previously.^40^ Briefly, the cells were incubated in DMEM medium containing 10 *μ*l of CCK-8 solution at 37 °C for 2.5 h. Then, the optical density (OD) at 450 nm was determined using a microplate reader (BIOTEK, Winooski, VT, USA), and the ratio of viable cells was calculated.

### Alizarin Red S staining

Cells were fixed in 4% paraformaldehyde for 10 min and rinsed three times with deionized water. The cells were then stained with 40 mM alizarin red S (Sigma), pH 4.0, for 10 min. Finally, the cells were rinsed three times with deionized water with gentle agitation.

### Real-time PCR

Total RNA from bone tissues or cells was prepared with TRIzol Reagent (Invitrogen). Complementary DNAs (cDNAs) were synthesized using the iScript cDNA Synthesis Kit (Bio-Rad, Hercules, CA, USA) according to the manufacturer's instructions. Quantitative real-time PCR was carried out using the ABI 7500 Sequence Detection System and SYBR Premix Ex Taq (Takara, Japan). The primer sequences are listed in Supplementary Table 1.

### Western blot analysis

Western blotting was performed as previously described.^41^ Total cell lysates were electrophoresed by SDS-PAGE and transferred to PVDF membranes (Millipore, Billerica, MA, USA). The membranes were incubated with specific antibodies to Runx2 (Abcam, Cambridge, MA, USA), *β*-catenin, p-ERK and ERK (Cell Signaling Technology, MA, USA) and then were reprobed with appropriate secondary antibodies labeled with IR dyes. The bands were detected using an Odyssey Infrared Imaging System (LI-COR Biosciences, Lincoln, NE, USA).

### ALP activity detection

Cells were washed twice with PBS and solubilized with lysis buffer (10 mM Tris-HCl (pH 7.5), 150 mM NaCl, complete protease inhibitor, and 1% NP-40). ALP activity was assayed using p-nitrophenylphosphate (Sigma-Aldrich) as a substrate. The protein content was measured using the BCA Protein Assay (Thermo Scientific, Rockford, IL, USA) according to the manufacturer's instructions. The ALP activity was expressed as Sigma unit/min/mg of protein.

### Enzyme-linked immunosorbent assay (ELISA)

Cells (1 × 10^4^ per well) were plated in six-well plates and subjected to the indicated treatment. On the following day, the medium was replaced with 2 ml per well of fresh DMEM containing 1% FBS, and the culture supernatants were collected after 24 h. The amounts of BSP were measured by ELISA using a Human BSP Quantikine ELISA Kit (R&D Systems, Minneapolis, MN, USA).

### Runx2 3'-UTR cloning and luciferase assay

To obtain the luciferase constructs, Runx2 mRNA 3'-UTRs containing the four miR-203-binding sequences were amplified by PCR. The primer sequences used in this experiment are listed in Supplementary Table 2. Next, different PCR-derived fragments from the Runx2 mRNA 3'-UTR were inserted into the pGL3 control vector (Promega, Madison, WI, USA) at the *Xba*1 site. The binding region mutations were obtained using a QuikChange Site-Directed Mutagenesis Kit (Stratagene, Santa Clara, CA, USA). Cells were seeded in six-well plates (1 × 10^6^ cells per well) and transfectedwith luciferase reporters. A dual-luciferase reporter assay system (Promega) was used to measure the reporter activity according to the manufacturer's instructions.

### Mouse experiments

The animal experimental protocols were approved by the Animal Research Committee of Shanghai Jiao Tong University Affiliated Sixth People's Hospital. The experiment was performed in accordance with approved guidelines. Four-week-old male BALB/c mice underwent an Achilles tenotomy (*n*=10 per group) with sharp dissection at the midpoint in the left leg. A 1-cm incision was made on the lateral aspect of the Achilles tendon to expose its full length. The Achilles tendon was then divided sharply at its midpoint with a surgical knife. The incision was closed with absorbable sutures. Next, the mice received an injection of agomiR-203, antagomiR-203 or their NCs (10 mg/kg). All of the mice were treated weekly with agomiR-203, antagomiR-203 or their NCs by injection at the local lesion in the tendon. All the mice were maintained for 8 weeks.

### Micro-CT analysis

Micro-computed tomography (micro-CT) was performed using a SkyScan (Kontich, Belgium) with a 9-*μ*m resolution, according to standard nomenclature. Briefly, the tibia was scanned using an X-ray source set at 60 kV with a 6-*μ*m pixel size. The region of interest (ROI) was defined to include the entire tibia, and two-dimensional (2D) image stacks were visually inspected to ensure that all the heterotopic bone was included within the ROI. Three-dimensional (3D) rendering was performed using built-in Scanco rendering software (Scanco Medical, Brüttisellen, Switzerland).

### Histology

At 8 weeks post-tenotomy, the animals were killed, and the tenotomized leg was fixed in 4% paraformaldehyde. HO tissues from patients were collected in surgical operations. All of these specimens were decalcified in a 10% EDTA solution for 1 month, embedded in paraffin and cut into 5-μm sections for staining. Immunohistochemical staining was carried out with primary antibodies against Runx2, *β*-catenin and p-ERK and with a 1:1000 dilution of an appropriate secondary antibody, and protein expression was visualized with a DakoCytomation Envision staining kit (Carpinteria, CA, USA).

### Statistical analyses

The data were represented as the means±S.D. Comparisons between groups were performed using Student's *t*-test, and a one-way ANOVA was used for multiple comparisons. All of the experiments were repeated at least three times, and representative experiments are shown. Differences were considered significant at *P*<0.05.

## Abbreviations

HO: heterotopic ossification
3'-UTR: 3'-untranslated regions
FOP: fibrodysplasia ossificans progressiva
ERK: extracellular signal-regulated kinase
OM: osteogenesis induction medium
ALP: alkaline phosphatase
OCN: osteocalcin
BSP: bone sialoprotein
MSCs: mesenchymal stem cells
NC: negative control
ELISA: enzyme-linked immunosorbent assay
DMEM: Dulbecco's Modified Eagle's Medium
CCK-8: cell count kit-8
