# Supplementary material for: miR-203 inhibits the traumatic heterotopic ossification by targeting Runx2
Source: Cell Death Dis. 2016 Oct 27;7(10):e2436–. doi: 10.1038/cddis.2016.325 (PMC5133990; doi:10.1038/cddis.2016.325)
Supplement: Supplementary Materials [file cddis2016325x1.pdf]

miR-203 inhibits the traumatic heterotopic ossification by targeting Runx2

Bing Tu

Shen Liu

Bo Yu

Jing Zhu

Hongjiang Ruan

Tingting Tang

Cunyi Fan

Supplementary Information

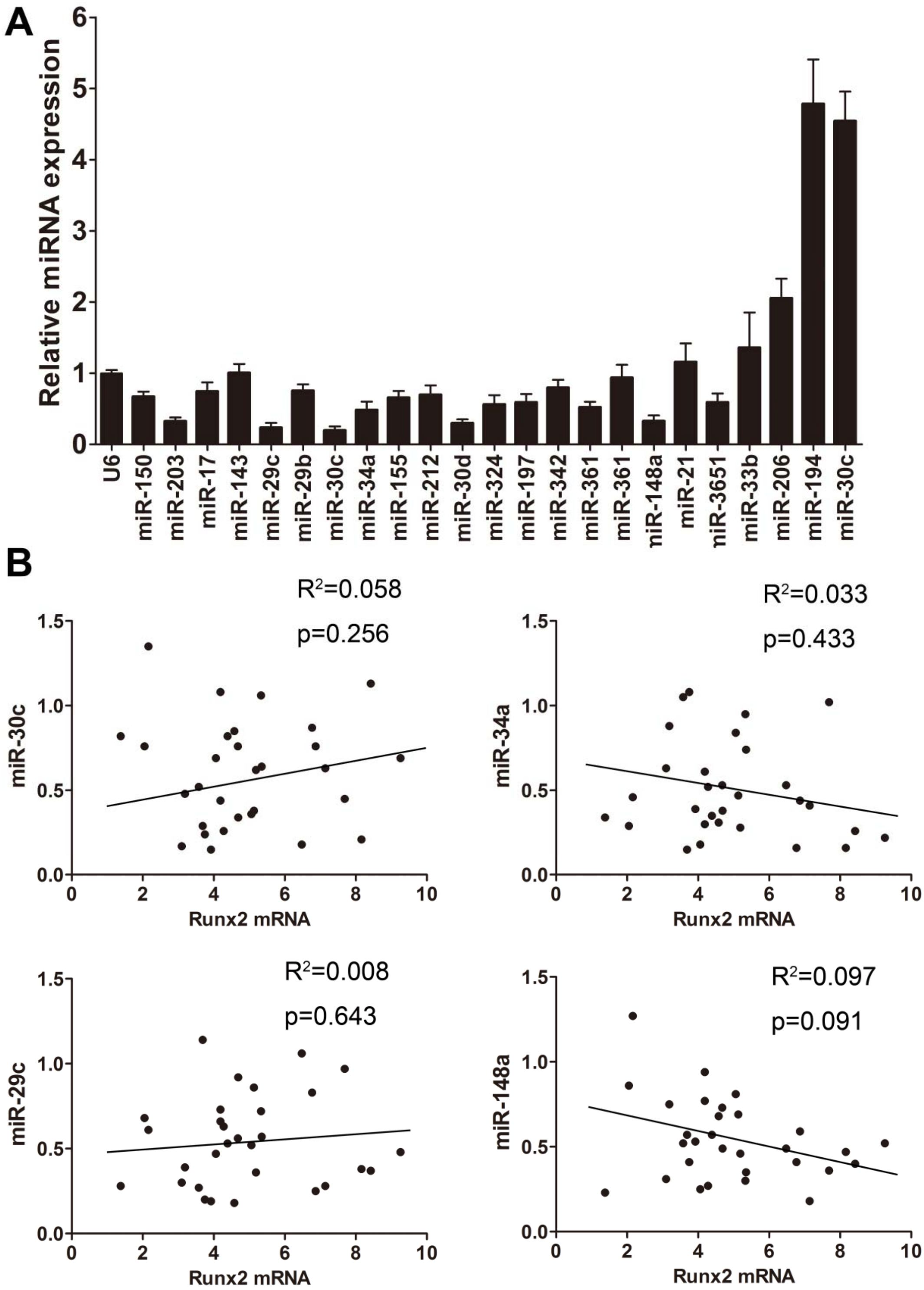

Supplementary Figure 1. miRNA screening and correlation analysis with Runx2 expression in human HO specimens. (A) Realtime-PCR analysis of miRNA expression (normalized by U6 in normal bones) in human HO. (B) Correlation analysis between miR-30c, miR-34a, miR-29c and miR-148a levels and Runx2 mRNA levels in human HO specimens.

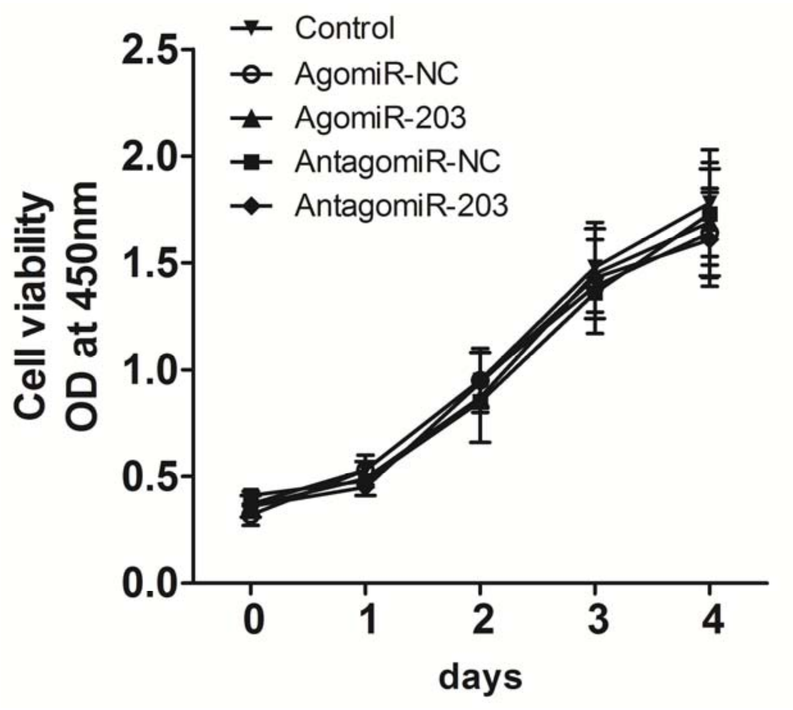

Supplementary Fig. 2. AgomiR-203 and antagomiR-203 have no effects on the proliferation of osteoblast cells. The hFOB1.19 osteoblast cells were transfected with agomiR-203 or antagomiR-203. The cell viability was examined by CCK-8 assay after 4 days. (n=3, p>0.05).

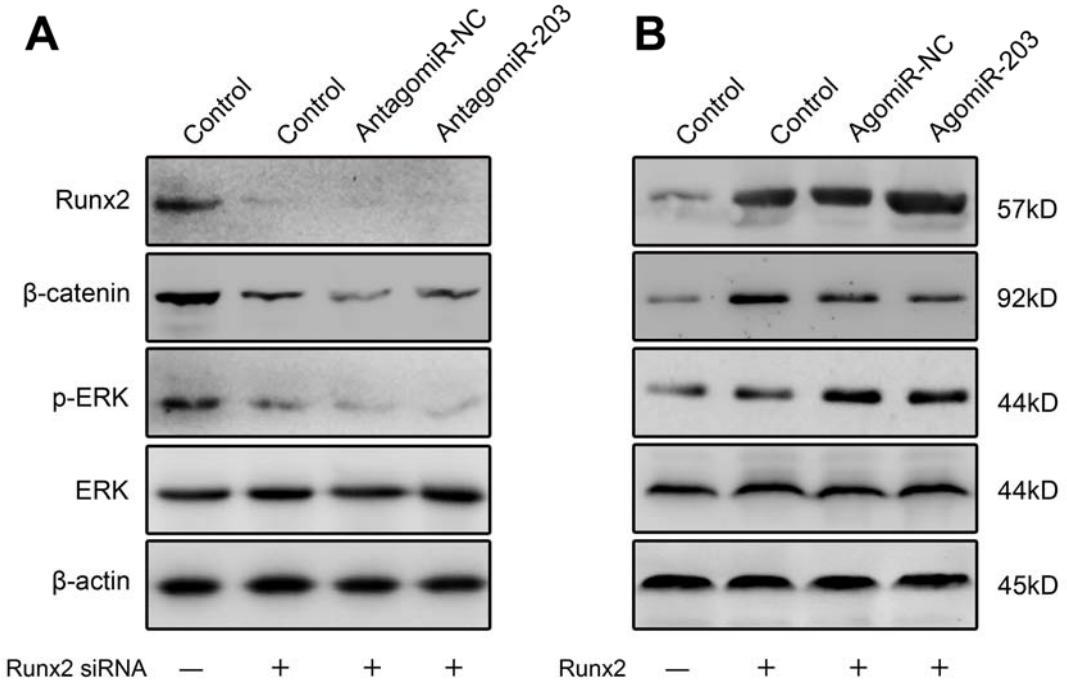

Supplementary Fig. 3. miR-203 inhibits the ERK and β-catenin signaling in a Runx2-dependent manner. (A) hFOB1.19 cells were co-transfected with Runx2 siRNA and antagomiR-203. Western blot analysis of the relative levels of Runx2, β-catenin, p-ERK and ERK protein expression at 72h. (B) hFOB1.19 cells were co-transfected with Runx2 overexpression plasmid and agomiR-203. Western blot analysis of the relative levels of Runx2, β-catenin, p-ERK and ERK protein expression at 72h.

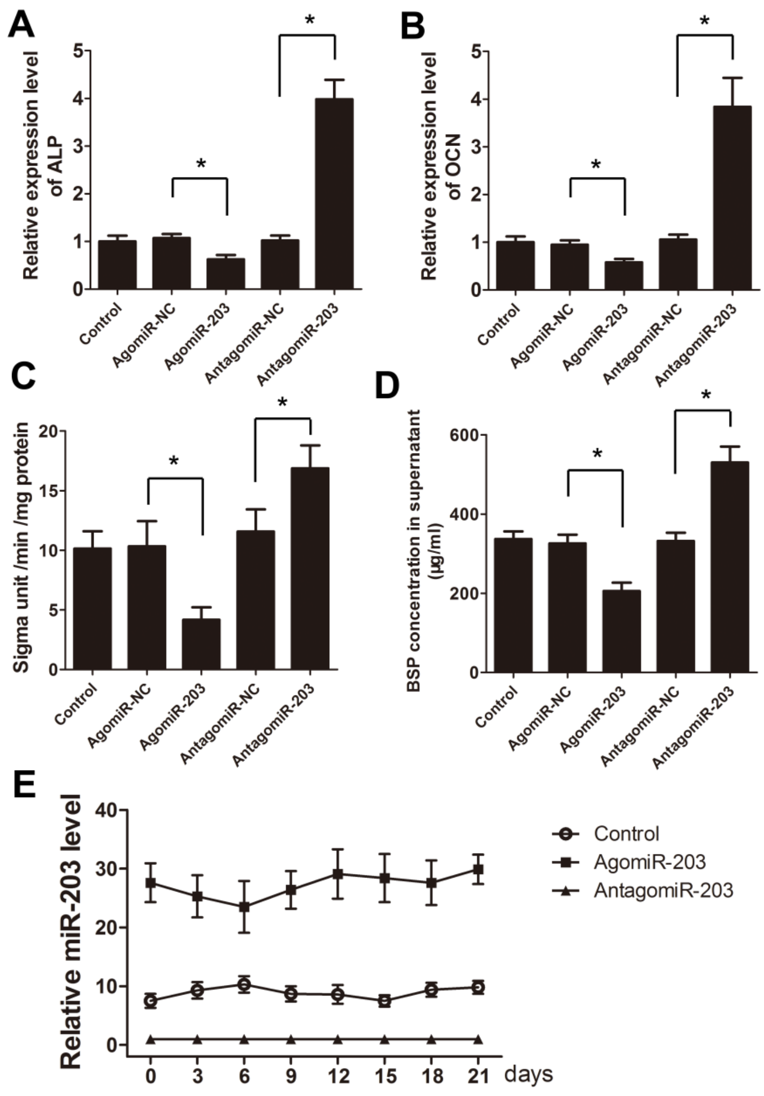

Supplementary Fig. 4. Effect of miR-203 on the activity of hFOB1.19 osteoblasts. (A) RT-PCR analysis of the miR-203 level in hFOB1.19 cells after treatment with 200 μM agomir-203, antagomir-203 or their NCs for 48 h. (B) RT-PCR analysis of OCN mRNA levels in hFOB1.19 cells after treatment with agomir-203, antagomir-203 or their NCs for 48 h. (C) ALP activity was examined in hFOB1.19 cells after treatment with agomir-203, antagomir-203 or their NCs for 48 h. (D) ELISA analysis of the BSP protein level in the supernatant of hFOB1.19 cells after treatment with agomir-203, antagomir-203

or their NCs for 48 h. (E) Time course of changes in the level of miR-203 as determined by RT-PCR analysis in hFOB1.19 cells after treatment with agomir-203 or antagomir-203 in OM. The data are representative of 3 independent experiments. The data are shown as the means ± SD. *$p<0.01$.

SUPPLEMENTAL TABLES:

Supplementary table 1. The RT-PCR primer sequences

Genes	Primer sequences
Runx2	forward 5'- CCGCCTCAGTGATTTAGGGC-3'
	reverse 5'- GGGTCTGTAATCTGACTCTGTCC-3'
β -catenin (CTNNB1)	forward 5'-AGCTTCCAGACACGCTATCAT-3'
	reverse 5'- CGGTACAACGAGCTGTTTCTAC-3'
ERK (MAPK1)	forward 5'-TCTGGAGCAGTATTACGACCC -3'
	reverse 5'- CTGGCTGGAATCTAGCAGTCT -3'
ALP	forward 5'- TGAGGGTGTGGCTTACCAG-3'
	reverse 5'- GATGGACGTGTAGGCTTTGCT-3'
OCN	forward 5'- CCTCACACTCCTCGCCCTATT-3'
	reverse 5'- CCCTCCTGCTTGGACACAAA-3'
GAPDH	forward 5'- ATGGGGAAGGTGAAGGTCG-3'
	reverse 5'- GGGGTCATTGATGGCAACAATA-3'
miR-203	forward 5'- GGGGTGAAATGTTTAGGAC-3'
	reverse 5'- CAGTGCGTGTCGTGGAGT-3'
U6	forward 5'-CTCGCTTCGGCAGCACA-3'
	reverse 5'-AACGCTTCACGAATTTGCGT-3'

Supplementary table 2. The primers used for cloning the pGL3-Runx2 3' UTR.

Gene	Primer sequence
Runx2 3'UTR-P1	forward 5'- TGCGTTTGGCTATGTGTTGT-3'
	reverse 5'- GGCAAAGCTGTGTTAGAGGC-3'
Runx2 3'UTR-P2	forward 5'- TTTGTAGGCCACCCAGCATT-3'
	reverse 5'- GCATCCCTAAAGTCACTCGGT-3'

Runx2 3'UTR-P3	forward 5'- GCAGCAACCCAGAAACACTT-3'
	reverse 5'- TGCTACAAAGAGAACCACGCT-3'
Runx2 3'UTR-P4	forward 5'- TGCACTGGGTCATGTGTTTG-3'
	reverse 5'- TCAAGGTTTGGAAGAAGTGTCT-3'
